# Novel candidate genes *AuxRP* and *Hsp90* influence the chip color of potato tubers

**DOI:** 10.1007/s11032-015-0415-1

**Published:** 2015-11-18

**Authors:** Dorota Sołtys-Kalina, Katarzyna Szajko, Izabela Sierocka, Jadwiga Śliwka, Danuta Strzelczyk-Żyta, Iwona Wasilewicz-Flis, Henryka Jakuczun, Zofia Szweykowska-Kulinska, Waldemar Marczewski

**Affiliations:** Plant Breeding and Acclimatization Institute, National Research Institute, Młochów, Platanowa 19, 05-831 Młochów, Poland; Department of Gene Expression, Institute of Molecular Biology and Biotechnology, Faculty of Biology, Adam Mickiewicz University, Umultowska 89, 61-614 Poznań, Poland

**Keywords:** *Auxin*-*regulated protein* (*AuxRP*) gene, DArT markers, *Heat-shock protein 90* (*Hsp90*) gene, Quantitative trait loci, Reducing sugar content, *Solanum tuberosum*

## Abstract

Potato (*Solanum tuberosum* L.) tubers exhibit significant variation in reducing sugar content directly after harvest, cold storage and reconditioning. Here, we performed QTL analysis for chip color, which is strongly influenced by reducing sugar content, in a diploid potato mapping population. Two QTL on chromosomes I and VI were detected for chip color after harvest and reconditioning. Only one region on chromosome VI was linked with cold-induced sweetening. Using the RT-PCR technique, we showed differential expression of the *auxin*-*regulated protein* (*AuxRP*) gene. The *AuxRP* transcript was presented in light chip color parental clone DG 97-952 and the RNA progeny of the bulk sample consisting of light chip color phenotypes after cold storage. This amplicon was absent in dark chip parental clone DG 08-26/39 and the RNA bulk sample of dark chip progeny. Genetic variation of *AuxRP* explained up to 16.6 and 15.2 % of the phenotypic variance after harvest and 3 months of storage at 4 °C, respectively. Using an alternative approach, the RDA-cDNA method was used to recognize 25 gene sequences, of which 11 could be assigned to potato chromosome VI. One of these genes, *Heat-shock protein 90* (*Hsp90*), demonstrated higher mRNA and protein expression in RT-qPCR and western blotting assays in the dark chip color progeny bulk sample compared with the light chip color progeny bulk sample. Our study, for the first time, suggests that the *AuxRP* and *Hsp90* genes are novel candidate genes capable of influencing the chip color of potato tubers.

**Electronic supplementary material:**

The online version of this article (doi:10.1007/s11032-015-0415-1) contains supplementary material, which is available to authorized users.

## Introduction

Glucose and fructose are osmotically active substances that protect plants during low temperature stress or act as cryoprotectants during frosts (Stitt and Hurry 2002). In potato (*Solanum tuberosum* L.) tubers, the content of the reducing sugars immediately after harvest is generally low. During tuber dormancy, sugar accumulates as a result of starch breakdown (Sonnewald and Kossmann 2013). This process depends on the genotype and reflects the environmental conditions of plant growth, the harvest date and the storage regimen (Tai and Coleman 1999; Jakuczun and Zimnoch-Guzowska 2004). Temperatures of 4–6 °C are the most favorable for tuber storage; however, these conditions stimulate the activity of amylolytic enzymes and the degradation of starch to sucrose. Sucrose can be transported into vacuoles or broken down into glucose and fructose. This phenomenon is known as cold-induced sweetening (CIS) (Isherwood 1973) and protects the plants from cold stress but is unacceptable to consumers. The content of reducing sugars in tubers for the processing industry should not exceed 0.07 % of fresh weight (FW) or 3.5 % of dry weight (DW) (McCann et al. 2010), because during processing reducing sugars participate in the Maillard reaction. The effect of the reaction can be seen as an undesirable brown coloration with an intensity that is proportional to the reducing sugar content. As a result, French fries and chips have an undesirable, bitter flavor. During CIS, the reducing sugar content can reach 2 % of the FW in tubers and significantly reduce their processing quality (Isherwood 1973). Therefore, low level of reducing sugars in potato tubers is one of the most important requirements in their processing worldwide.

Several enzymes involved in starch synthesis and breakdown, glycolysis and hexogenesis have been identified and characterized at the biochemical and molecular levels in potato tubers (Sowokinos 2001; Solomos and Mattoo 2005; Lin et al. 2015). These data, together with knowledge about the intracellular partitioning of metabolites (Malone et al. 2006; Farre et al. 2008), has led to the conclusion that the biochemistry of carbohydrate metabolism in potato tubers is well studied. However, the genetic and metabolic regulation of this process is still unclear. Reducing sugars content in potato tubers is a quantitative trait, with heritabilities ranging from 0.47 to 0.98 (Cunningham and Stevenson 1963; Pereira et al. 1994; Jakuczun and Zimnoch-Guzowska 2004; Hamernik et al. 2009). Quantitative trait loci (QTL) analyses (Douches and Freyre 1994; Menendez et al. 2002; Werij et al. 2012) as well as the construction of the potato molecular function map (Chen et al. 2001) represent milestones in the application of a candidate gene approach to assess DNA variation of genes important for carbohydrate metabolism and transport in potato tubers. These findings revealed that different sets of genes may control the synthesis of reducing sugars in tubers at harvest, after cold storage and after reconditioning (controlled tuber warming). Recently, natural DNA variation in several genes that function in the starch–sugar interconversion was found to be associated with tuber quality traits (Schreiber et al. 2014). The loci on chromosomes IX and X encoding apoplastic, cell-wall-bound isoforms of the acid invertase (Li et al. 2005, 2008; Draffehn et al. 2010) and the locus on chromosome III encoding an intra-cellular, soluble acid invertase (Draffehn et al. 2010; Li et al. 2010, 2013) were described as the most promising genetic factors associated with chip quality in tetraploid potatoes. The high flexibility of carbon metabolism depends on the physiological state, environmental conditions and sequence diversity. The DNA polymorphisms in the candidate genes (single nucleotide polymorphisms, insertion or deletion polymorphisms) with potential influence on their functions additionally complicate the identification of key genetic factors involved in cold sweetening. Comparative proteomics (Fischer et al. 2013) and mapping of expression QTL (e-QTL) (Kloosterman et al. 2012) have created new possibilities for the recognition of factors involved in the accumulation of reducing sugars in potato tubers.

The objective of this study was to identify candidate genes that affect the chip color of potato tubers in interspecific *Solanum* diploid hybrids. We applied two alternative approaches to identify novel genetic factors. The first method was based on QTL mapping, followed by a reverse transcription polymerase chain reaction (RT-PCR). The second method was based on transcriptome investigation by representational difference analysis of cDNA (RDA-cDNA), followed by RT-quantitative PCR (RT-qPCR) and western blotting analysis. We have successfully used the RDA-cDNA technique for the identification of genes specifically expressed in a liverwort *Pellia endiviifolia* male and female thalli producing antheridia and archegonia, respectively (Sierocka et al. 2011, 2014). Here, combining information from genetic studies and gene/protein expression data may yield a more accurate picture of genetic processes underlying complex traits than that obtained by using them separately (Pérez-Enciso et al. 2003).

## Materials and methods

### Plant material

The diploid potato (*S. tuberosum*) parental clones DG 97-952 and DG 08-26/39 were crossed in 2010. Both parents were interspecific, multigenerational *Solanum* hybrids originating from *S. tuberosum*, *S. acaule*, *S. chacoense, S. demissum*, *S. gourlayi*, *S. microdontum*, *S. phureja*, *S. stenotomum*, *S. verrucosum* and *S. yungasense*. The theoretical input of the *Solanum* spp. to the genetic composition of the parents was calculated based on the pedigree data. The percentage of *S. tuberosum* in the origin of DG 97-952 and DG 08-26/39 were 67 and 68 %, respectively. Values for chip color after harvest of the parental clones DG 97-952 and DG 08-26/39 were 8.5 and 4.5, respectively. The parental clones and 92 F1 individuals (population 11–40) were used for DArT map construction and QTL analysis. Plants were first sprouted for 2 weeks in the sprouting chamber and then planted in tents where they were grown from May to October (average: 18 weeks). Three replications of the parents and each of the progeny were grown in the tents in 2011 (seedlings) and in 2012–2013 (first and second tuber generations).

### Chip color assessment

The chip color for tubers of the parental clones and F1 individuals was evaluated in three subsequent years (2011, 2012 and 2013). Tubers harvested in 2011 were assessed in one batch 2 weeks after harvest (AH). Tubers harvested in 2012–2013 were fried in three batches each in three replications per genotype. The first batch was fried 2 weeks after harvest, the second batch 3 months after cold storage at 4 °C (CS) and the third batch 2 weeks after reconditioning at 19 °C (RC). For each replication, four slices of each of two potato tubers were fried. The frying color was visually assessed on a scale from 1 (dark) to 9 (light) as described by Jakuczun et al. (1995). Cultivars Pasat and Saturna were used as dark- and light-colored standards, respectively.

### Genetic mapping and QTL analysis

Genomic DNA of parental clones and F1 individuals was isolated from leaves using the DNeasy Plant Maxi kit (Qiagen GmbH, Hilden, Germany) according to manufacturer’s protocol. DArT analysis was performed using the Diversity Array Pty Ltd. Canberra, Australia, as described for *S. michoacanum* and *S. ruiz*-*ceballosii* by Śliwka et al. (2012a, b), following the protocols previously developed for other plant species (Jaccoud et al. 2001; Wenzl et al. 2004). The genetic map was enriched in sequence-specific CAPS (cleaved amplified polymorphic sequence) and SCAR (sequence characterized amplified regions) markers (Supplementary Table 1). The PCR mixture (20 μl) contained 1× DreamTaq buffer, 0.25 mM of each dNTP, primers (0.25 μM), DreamTaq Polymerase (0.01 U, Fermentas) and DNA. The PCR parameters were initial denaturation at 94 °C for 60 s, followed by 40 cycles of denaturation at 93 °C for 25 s, annealing at 48–62 °C (Supplementary Table 1) for 35 s and extension at 72 °C for 90 s with a final extension at 72 °C for 5 min. The PCR products were digested and visualized in a 1.2 % agarose/TBE (100 mM Tris–HCl, 83 mM boric acid, 1 mM EDTA, pH 8.4) gel containing 0.5 μg ml^−1^ ethidium bromide. Linkage analysis was performed as described previously using JoinMap^®^ 4 (Van Ooijen, 2006) with the following settings: CP population type (first creating maternal and paternal linkage maps and then creating a common population map), independence LOD as a grouping parameter (linkages with LOD > 3 were considered significant), regression mapping algorithm and Haldane’s mapping function (Śliwka et al. 2012a). The obtained linkage groups were oriented and named (chromosomes: I–XII) by comparison with earlier DArT maps of related species (Sharma et al. 2013; Śliwka et al. 2012a, b). Interval mapping of the QTL was performed using MapQTL^®^ 6 (Van Ooijen 2009). QTL with LODs equal or exceeding 3 were treated as significant.

### RNA isolation and construction of RNA and cDNA bulk samples

RNA was isolated from tubers harvested in 2012 (first tuber generation) after 3 months of cold storage at 4 °C. Parental clones DG 97-952 and DG 08-26/39 and ten F1 individuals, each in two replications, characterized by different chip color (5 with chip color 1–3 and 5 with chip color 8–9) were used for RNA isolation according to the protocol of Chomczyński and Sacchi (1987) using the TRIZOL reagent. Briefly, 1 g of frozen tubers were ground in liquid nitrogen prior to the addition of 4 ml of TRIZOL reagent. After incubation at room temperature and centrifugation, the supernatants were transferred to fresh tubes. The extraction was performed twice in 3 ml of chloroform. The RNA was precipitated 15 min after the addition 0.6 ml of salt solution (0.8 M sodium citrate and 1.2 M sodium chloride) and 0.6 ml of isopropanol. The RNA concentration and quality were determined using a biophotometer (Eppendorf) at 260, 280 and 230 nm. Two types of bulk samples were prepared. For bulk samples L^I^ and D^I^, reverse transcription of RNA (2 μg) from each genotype was performed using the RevertAid kit (Fermentas) according to the manufacturer’s protocol. The cDNA from five genotypes (in two replications) that were characterized by light chip color (color 8–9) and five that were characterized by dark chip color (color 1–3) were mixed together at equal amounts to form the bulk samples L^I^ and D^I^, respectively. For the bulk samples L^II^ and D^II^, equal amounts (60 µg) of the RNA progeny samples were mixed together to prepare the bulk samples.

### Expression of the *AuxRP* gene

The cDNA of the bulk samples L^I^ and D^I^ was used for PCR amplification (Supplementary Table 1). The PCR mixture (20 μl) contained 1× DreamTaq buffer, 0.25 mM of each dNTP, primers (0.25 μM), DreamTaq Polymerase (1 U, Fermentas) and cDNA. Water was used as a negative control. The PCR products were confirmed using electrophoresis in 1 % agarose/TBE gels with ethidium bromide (Midi Horizontal Electrophoresis Unit Set, Thermo Scientific). All reactions were performed using the G-STORM thermal cycler (Gene Technologies, UK) and visualized using transilluminator UV (Vilber Lourmat, Germany). The PCR products for *AuxRP* (PGSC0003DMT400077929) when cDNA was used as a template were cloned in pGEM-T vector in the Laboratory of DNA Sequencing and Oligonucleotides Synthesis IBB PAS, Warsaw. After cloning, 96 clones were sequenced. Consensus sequences were obtained using blastn algorithm available at the Potato Genomics Resource platform (http://solanaceae.plantbiology.msu.edu). Sequence-specific primers were constructed based on the cloned sequences. Specific primers were developed as follows: AuxRP forward: 5′-AAGGCGGACGGAAAAGTAATCT-3′ and AuxRP reverse: 5′-CAAGTTCAAGCAAGTCCATC-3′. The expression levels of *AuxRP* were measured on cDNA from the bulk samples L^I^ and D^I^ using the RT-PCR method. Experiment was repeated at least five times, and data obtained from representative, individual experiments were presented.

### Representational difference analysis of cDNA (RDA-cDNA) procedure

The poly(A)^+^ RNA was isolated from 600 µg of total RNA from bulk samples L^II^ and D^II^ using the PolyATract mRNA Isolation System (Promega, No. Z5300) according to manufacturer’s protocol. Double-stranded cDNA (ds-cDNA) was synthesized using the cDNA Synthesis System (ROCHE, No. 11117831001) according to manufacturer’s protocol. The oligonucleotides utilized in RDA-cDNA were published by Hubank and Schatz (1994). The ds-cDNAs (1.5 μg) of the TESTER and the DRIVER were used to generate amplicons. RDA-cDNA was conducted in two directions, each in independent experiments: ds-cDNA from genotypes with a light chip color was used once as the TESTER and the second time as the DRIVER. Four rounds of subtractive hybridization (SH^I^–SH^IV^) were performed using the quantitative TESTER to DRIVER ratios as follows: 1:100 for the first round, 1:800 for the second round, 1:400,000 for the third round and 1:1,000,000 for the final round. Hybridization was performed at 67 °C for 48 h. After each round of hybridization, the RDA-cDNA products were separated by electrophoresis in 1.5 % agarose gels (Midi Horizontal Electrophoresis Unit Set, Thermo Scientific). The final product of the fourth hybridization round was sent to the Laboratory of DNA Sequencing and Oligonucleotides Synthesis IBB PAS, Warsaw, for cloning and sequencing. Sequences of the SH^IV^ products were analyzed using the blastn algorithm available at the Potato Genomics Resource platform (http://solanaceae.plantbiology.msu.edu/).

### RT-qPCR analysis of the *Hsp90* gene selected by RDA-cDNA

Validation of gene expression derived after subtractive hybridization was performed using the RT-qPCR method. The same RNA isolated during the RDA-cDNA experiment (tuber after CS) was used. Two micrograms of each RNA sample from the parents and F1 individuals were reverse transcribed into cDNA using the RevertAid kit (Thermo Scientific, no. K1691) with Random Hexamer primers. Equal amounts of the cDNA of ten F1 individuals were mixed (two replications each; five characterized by light chip color and five characterized by dark chip color). For RT-qPCR, 50 ng of each bulk and parental genotype was collected. Analysis was performed using the LightCycler^®^ 480 SYBR Green I Master (Roche), and ∆∆*C*_t_ values were calculated. Specific primer pairs were designed on the basis of the cloned amplicon *Hsp90* (PGSC0003DMT400074377) derived from the RDA-cDNA experiment: forward primer Hsp90a 5′-GTTCCCTTGCTTTTTGAGACCG-3′ and reverse primer Hsp90a 5′-GGGAACTCCAATGCAGGCGTG-3′. *α*-*tubulin* was used as the reference gene with the following primers pairs: 57-α-tubulin forward 5′-AATTTGTCGACTGGTGTCCT-3′ and 57-α-tubulin reverse 5′-GTCAATGCGAGAGAAGACCT-3′ (Śliwka et al. 2013). The following program was applied: denaturation at 95 °C for 5 min; 40 amplification cycles of 95 °C for 10 s, 65 °C for 20 s and 72 °C for 30 s. Then, PCR product melting was performed in a temperature range of 65–97 °C, and the melting curve was analyzed to confirm the amplification of gene-specific products. A single peak on the melt curve analysis indicated the presence of the single PCR product. The results were expressed as relative expression levels from three independent biological experiments, with four replications in each set.

### Western blot analysis of Hsp90

Total proteins were isolated from tubers of the parental clones and ten genotypes differing in chip color (chip color 1–3 and 8–9) each in two replications (the same genotypes used in the RDA-cDNA and gene expression experiments) after storage at 4 °C in 2012. Protein isolation was performed separately for each clone according to the protocol of Urbany et al. (2012). Briefly, 250 mg of potato tubers were ground in liquid nitrogen and extracted in extraction buffer (2 % SDS; 0.1 % Triton X-100; 10 mM EDTA; 25 mM DTT; 30 % sucrose; and 0.05 M TRIS/HCl, pH 8.0). After incubation on ice, the extract was treated with basic phenol. The upper phase was transferred to fresh tubes and washed with extraction buffer. Proteins were precipitated in 0.1 M ammonium acetate in methanol at −20 °C. After centrifugation, the proteins were washed first with acetone, second with methanol, and then solubilized in a 2 M thiourea/7 M urea solution. Equal amounts of proteins from genotypes with light or dark chip colors were mixed and grouped into the protein bulk samples L^II^ and D^II^. A total of 5 µg of total proteins from each sample were resolved by electrophoresis on a 10 % SDS-PAGE gel and subjected to electrotransfer onto a nitrocellulose membrane (GE Healthcare, 0.45 µm). Then, the nitrocellulose was incubated in 5 % non-fat dry milk powder in T-TBS buffer (0.05 M Tris–HCl, 0.15 M NaCl, and 1 % Tween, pH 7.6) for 1 h. After blockage of nonspecific protein binding, the nitrocellulose was incubated with the specific antibody anti-Hsp90-1 (Agrisera, No. AS08 346) or anti-Hsp90-2 (Agrisera, AS11 1629) in T-TBS with 2 % dry milk overnight (both diluted 1:3,000). Next, the membrane was incubated with a secondary antibody (goat anti-rabbit IgG, Agrisera, AS09 602) conjugated with horseradish peroxidase in T-TBS with 2 % dry milk diluted 1:25,000 for 2 h. For color development, DAB (3,3′-diaminobenzidine solution, Sigma Aldrich, No. D3939) was added to the membrane and incubated for 2 min. The Western analysis was performed for four independent experiments.

## Results

### Chip color after harvest, cold storage and reconditioning

Chip color measured after harvest (AH), cold storage (CS) and reconditioning (RC) in the parents DG 97-952 and DG 08-26/39 was 8.5 and 4.5; 7.0 and 4.0; 6.7 and 5.3, respectively. The corresponding values for F1 individuals from mapping population 11–40 are presented in Supplementary Table 2. After harvest, 66 genotypes had chip color ranging from 4 to 6. Cold storage resulted in higher concentrations of reducing sugars in 79 % of genotypes. The most frequent genotypes (45 %) had chip color 3–4, with the simultaneous appearance of genotypes with chip color 1–2 (17 %). The number of genotypes with chip color 1–3 increased during RC (8 % of genotypes became darker after RC compared with CS). After reconditioning process genotypes with chip color scores 2–4 were the most frequent (71 %). The distribution of data was normal for chip color after AH and deviated from normality for CS and RC (Supplementary Fig. 1).

### Major QTL for chip color are localized on chromosomes I and VI

Among the 3331 DArT markers scored in population 11–40, 2089 markers were segregated (i.e., were present in more than 10 % and less than 90 % of progeny individuals). We excluded from analyses markers with more than 10 % missing data (172 markers) and those with unknown origins (parental clone data missing, 130 markers). Further markers were removed on the basis of the DArT quality parameter call rate <85 (3 markers), resulting in 1784 remaining markers. Markers with identical patterns of segregation were excluded by the JoinMap^®^ 4 program. The final genetic map consisted of 1420 markers, including 1410 DArT markers and 8 CAPS and 2 SCAR markers. A total of 370 markers originated from parent DG 97-952 and 490 from DG 08-26/39, while 560 markers descending from both parents. The total length of the map reached 1000.2 cM. The numbers of markers located on particular chromosomes varied from 29 on chromosome IV to 226 on chromosome IX. The lengths of the chromosomes ranged from 55 (for VI) to 108 cM (for II) (Supplementary Fig. 2).

The QTL for chip color were detected on chromosomes I and VI (Table 1, Supplementary Fig. 3). The QTL on chromosome I was significant in the AH and RC datasets, while the QTL on chromosome VI was significant for all three datasets (AH, CS and RC). The QTL for chip color after CS was detected only on chromosome VI. The effect of the QTL on the mean chip color after AH reached 17.5 % (LOD 3.84) of the variance explained by the QTL on chromosome I and 18.3 % (LOD 4.03) by the QTL on chromosome VI. Marker alleles on chromosomes I and VI affecting the trait descended from DG 08-26/39. The major QTL for mean chip color after CS was mapped onto chromosome VI between 71.1 and 83.7 cM and explained up to 23.5 % of the variation. The most significant QTL for chip color after RC was detected on chromosome VI RC12 and explained 24.0 % of the variance (LOD 5.47); this QTL descended from DG 97-952 and DG 08-26/39. The impacts of the QTL on mean chip color after RC on chromosome I and VI was 17.5 % at LOD 3.83 and 3.84, respectively. The QTL effect varied between data sets over the time course of the study, indicating the significance of the environment during growing season and genotype × environment interaction on the sugar metabolism later in the storage. Most of the QTL for chip color after CS on chromosome VI were significant in all data sets (CS12–CS13 and mean CS) (Table 1), indicating that this trait was stable in different vegetation seasons. The QTL on chromosome I was significant in three out of four datasets obtained after harvest (AH12, AH13 and mean AH), while the QTL on chromosome VI was detected in a different combination of three AH datasets (AH11, AH12 and mean AH). The least stable trait was chip color after RC; the significant QTL were detected only for the mean RC on chromosome I and RC12 and mean RC on chromosome VI (Table 1).

**Table 1:** QTL detected for mean (all years of testing) chip color after harvest (AH), cold storage (CS) and re-conditioning (RC) as well as in particular seasons 2011–2013 (e.g., AH11–AH13) in the diploid potato population 11–40

Chromosome	Trait	Marker at peak or markers flanking virtual peak interval	Parent^a^	LOD	*R* ^2^ (%)	Peak position (cM)	Significant interval (cM)
I	AH12	pPt-473623-pPt656201	P2	3.59	16.4	48.3	45.7–49.7
	AH13	pPt-654681	P1, P2	3.60	17.4	5.5	4.7–5.9
	AH	pPt-473623-pPt656201	P2	3.84	17.5	48.3	44.7–50.7
	RC	pPt-536041	P2	3.83	17.5	67.8	66.0–72.9
VI	AH11	pPt-540198-Myb48g	P2	4.15	19.0	73.9	71.1–75.7
	AH12	pPt-536863	P1, P2	4.12	18.6	75.9	71.7–72.3 75.8–79.0
	AH	pPt-538874	P2	4.03	18.3	71.3	71.1–73.9 75.8–80.2
	CS12	pPt457555-ppt-471500	P2	5.27	23.2	77.7	54.7–60.7 70.1–74.6 75.7–83.7
	CS13	pPt-457555	P2	4.72	22.1	76.7	72.1 75.8 76.3–78.1
	CS	pPt457555-ppt-471500	P2	5.36	23.5	77.7	71.1–83.7
	RC12	pPt-536863	P1, P2	5.47	24.0	75.9	75.7–79.4
	RC	pPt-536863	P1, P2	3.84	17.5	75.9	75.8–75.9

Interval mapping of QTL was performed using MapQTL^®^ 6 (Van Ooijen 2009)

^a^P1—inherited from DG 97-952; P2—inherited from DG 08-26/39

### CAPS and SCAR markers on chromosome VI

The genetic map of the DArT markers was enriched with eight CAPS and two SCAR markers which were selected from the QTL region on chromosome VI (Supplementary Table 1), of which nine were developed based on DArT sequences or gene sequences available at the Potato Genomics Resource (PGSC) platform within chromosome VI. One DNA marker for the locus *Hsp90* was developed using the sequences identified in the RDA-cDNA experiment. Eight and two markers originated from DG 97-952 and DG 08-26/39, respectively. Five of the markers showed linkage to chip color in at least two datasets. The DNA markers AuxRP, Chaperone DnaJ, Myb48d and Zinc were linked with chip color in the AH and CS samples, while the marker Nod was linked with chip color in the CS and RC samples. The markers 965p2, Myb48 g and pPt874a were significantly linked only to CS. The most significant CAPS markers for chip color AH were derived from the loci *AuxRP* and *Myb48d*; the same phenotype effects explained 16.6 % of the variance (LOD 3.62). For CS, Nod explained 20.7 % of the variance (LOD 4.64); moreover, it was the only marker allele with significant effect on RC, where it explained 14.8 % of the phenotypic variance (LOD 3.20) (Table 2).

**Table 2:** Locations and effects on phenotypic traits, chip color after harvest (AH), cold storage (CS) and re-conditioning (RC) of the PCR markers from chromosome VI

Locus	Function assignment	Parent^a^	Position (cM)	Effect					
				AH		CS		RC	
				LOD	*R* ^2^ (%)	LOD	*R* ^2^ (%)	LOD	*R* ^2^ (%)
*Hsp90*	Heat-shock protein	P1	30.0						
*pPt837b*		P1	48.3						
*Myb48* *g*	Transcription factor Myb48	P2	74.6			3.18	14.7		
*Myb48d*	Transcription factor Myb48	P1	75.9	3.62	16.6	3.29	15.2		
*AuxRP*	Auxin-regulated protein	P1	75.9	3.62	16.6	3.29	15.2		
*Nod*	Nodulin 26	P2	76.3			4.64	20.7	3.20	14.8
*Zfp*	Zinc finger protein	P1	79.4	3.51	16.1	3.91	17.8		
*Chaperone DnaJ*	Chaperone DnaJ (Hsp40)	P1	79.4	3.52	16.1	3.91	17.8		
*pPt874a*		P1	80.6			3.80	17.3		
*965p2*		P1	80.9			3.75	17.1		

Only significant LOD and *R* ^2^ values are listed

^a^P1—inherited from DG 97-952; P2—inherited from DG 08-26/39

### *AuxRP* expression

**Fig. 1:**
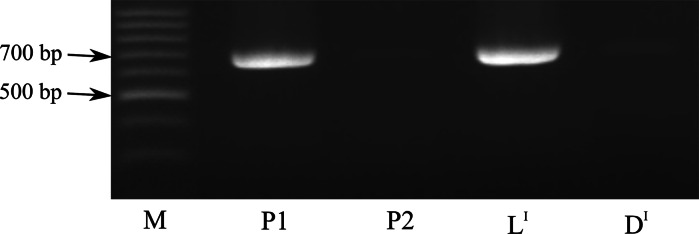
Expression profiling of *AuxRP* in parental clones P1 (DG 97-952, chip color 7) and P2 (DG 08-26/39, chip color 4), and bulk samples L^I^ (chip color 8–9) and D^I^ (chip color 1–3) after cold storage (CS). M—molecular weight marker (100-bp DNA ladder)

The expression of four genes mapped onto chromosome VI. *Chaperone DnaJ*, *Myb transcription factor* (*Myb48*), *zinc finger protein* (*Zfp*) and *auxin*-*regulated protein* (*AuxRP*) was assessed using the RT-PCR technique. Only one gene (*AuxRP*) was shown to be expressed differentially in the light and dark chip samples after CS. The specific amplicon was 660 bp long and was expressed only in DG 97-952 and bulk L^I^ with light chip color phenotypes after cold storage (Fig. 1). Linkage to a QTL for chip color and differential expression indicated that *AuxRP* may be a candidate gene influencing the chip color of potato tubers.

### Evaluation of differentially expressed genes

**Fig. 2:**
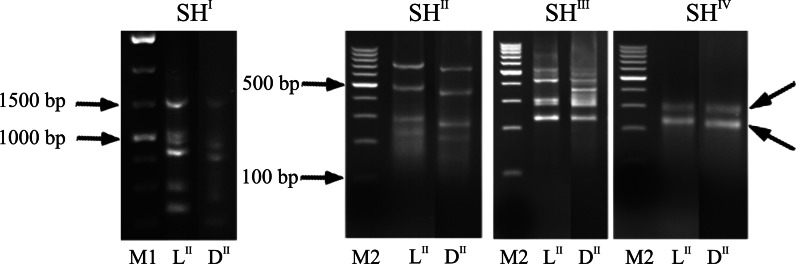
RDA-cDNA amplicons in 1.5 % agarose gels after the first (SH^I^), second (SH^II^), third (SH^III^) and fourth (SH^IV^) rounds of subtractive hybridization. The 250- and 300-bp fragments selected in the SH^IV^ round are marked by *arrows*. M1—1-kb DNA ladder, M2—100-bp DNA ladder, L^II^—genotypes with light chip color as a TESTER, D^II^—genotypes with light chip color as a DRIVER

The RDA-cDNA technique was employed to identify candidate genes responsible for reducing sugar accumulation in potato tubers stored at 4 °C. The cDNA obtained from mRNA isolated from genotypes with light chip color after CS was used once as the TESTER (L^II^) and once as the DRIVER (D^II^). After the fourth round of hybridization (SH^IV^), two distinct bands at 250 bp and 300 bp were obtained from the L^II^ and D^II^ samples, respectively (Fig. 2). The cDNA fragments were cloned and sequenced. Of the 109 clones from L^II^ and D^II^, 41 different sequences were obtained, in which 24 came from bulk L^II^ and 17 from bulk D^II^ (Supplementary Table 3). Among them, 16 and 9 sequences were assigned to protein coding genes in the bulk samples L^II^ and D^II^, respectively. Sequences that could be located to chromosome VI represented 27 % of the gene sequences obtained from the RDA-cDNA (Supplementary Table 3). To confirm which of these genes were characteristic for bulk L^II^ and D^II^, RT-PCR was performed with the same RNA template used for the RDA-cDNA experiment.

### *Hsp90* gene expression and Hsp90 protein expression

**Fig. 3:**
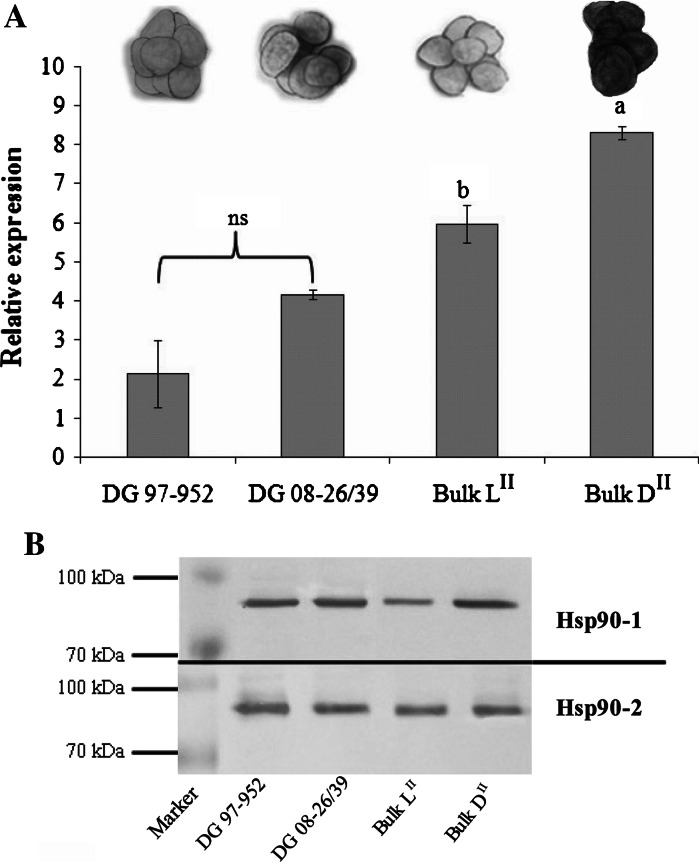
**a** Expression level of the *Hsp90* gene in tubers after cold storage (CS); *ns* non-significant differences between parental clones (*Student’s t test*); *a* and *b* homogenous groups between bulk samples L^II^ and D^II^ (*Tukey’s test*). **b** Amounts of Hsp90-1 and Hsp90-2 protein isoforms in tubers after cold storage (CS); Marker—Prosieve^®^ QuadColor™ Protein Marker. Parental clones: DG 97-952 (chip color 7) and DG 08-26/39 (chip color 4); bulk samples: L^II^ (chip color 8–9) and D^II^ (chip color 1–3)

Among the candidate genes selected in the RDA-cDNA experiment, we found that only *Hsp90* was informative and differentially expressed in bulk samples L^II^ and D^II^. The cDNA sequences of *Hsp90* found in bulk samples L^II^ and D^II^ were 240 bp and 154 bp long, respectively, and had 95 % similarity (*E* value = 6e − 70) on the 154-bp cDNA fragment. To confirm this finding and determine which of these alleles were characteristic for bulk L^II^ and D^II^, specific primer pairs for both sequences were designed. Expression of *Hsp90* (originated from bulk D^II^), determined by RT-qPCR, was not significantly different in the parents DG 97-952 and DG 08-26/39. However, the expression level of *Hsp90* in bulk D^II^ was significantly higher than in bulk L^II^ (Fig. 3a).

The protein expression levels of the Hsp90-1 and Hsp90-2 isoforms were detected by western blotting. Hsp90-2 is the constitutive isoform, and its expression did not vary between the parental clones and the bulk samples (Fig. 3b). Hsp90-1 is the isoform involved in responses to biotic and abiotic stresses (Kadota and Shirasu 2012; Xu et al. 2012). In this case, the amounts of the protein in the parental clones were similar. The amount of Hsp90-1 protein in bulk D^II^ was significantly higher compared with bulk L^II^ (Fig. 3b). Bulks L^II^ and bulk D^II^ were constructed for tubers consisting very low (chip color 8–9) and very high (chip color 1–3) content of reducing sugars, respectively (Fig. 3a). Thus, the higher expression level of stress-induced Hsp90-1 isoform as well as the higher expression of *Hsp90* gene in bulk D^II^ indicates that this gene may have an effect on reducing sugar content in potato tubers.

## Discussion

Between two and six QTL for chip color have been detected in previous studies. Six QTL for chip color (two on chromosome II, one on IV, two on V, and one on X) were identified in a population of the diploid hybrid of *S. tuberosum* and *S. chacoense* after storage at 10 °C for 45 days (Douches and Freyre 1994). Menendez et al. (2002) revealed six QTL explaining more than 10 % of the variability in reducing sugars that were located on chromosomes I, III, VII, VIII, IX, and XI in diploid *S. tuberosum* tubers stored at 4 °C for 3 months. Recently, two (on linkage groups IX and X), four (on linkage groups III, V, VIII and X) and two (on linkage groups V and X) QTL for chip color were detected in diploid potato clones of *S. phureja, S. vernei* and *S. tuberosum* origin after harvest, cold storage and reconditioning, respectively (Werij et al. 2012). In our study, two QTL on chromosomes I and VI that were linked with chip color were detected both at harvest and after reconditioning. We identified only one QTL for chip color after cold storage, located at the end of the long arm of chromosome VI. From three previous chip color/reducing sugar QTL linkage mapping studies (Douches and Freyre 1994; Menendez et al. 2002; Werij et al. 2012), one (Menendez et al. 2002) has reported 3 sugar QTL on chromosome VI: Sug6a, Sug6b and Sug6c. In the recent paper (Schreiber et al. 2014), invertases and hexokinase have been proposed to contribute to the effect of chromosome VI on chip color.

The genes *Zfp*, *Myb48*, *Nodulin 26* (*Nod*) and *Chaperone DnaJ* that were mapped to chromosome VI showed QTL effects at different significance levels in population 11–40. They accounted for 14.7–20.7 % of the phenotypic variance. *Myb48* is a member of the *Myb* superfamily of sequence-specific transcription factors; the members of this family are recognized as genetic factors associated with increasing tolerance to abiotic stresses in plants (Dubos et al. 2010; Shin et al. 2011). *Nod* encodes an aquaporin involved in selective water transport that allows the regulation of osmotic pressure and adjustment of osmotic potential. Eight aquaporins have been localized on potato chromosome VI, including the gene *St*-*TIP1;2* (Venkatesh et al. 2013). This gene corresponds with the map position of *Nod* detected in our study. The gene *Zfp* belongs to a large family of transcription factors that can play various regulatory roles in plants, including abiotic stress responses (Giri et al. 2011).

We used two alternative approaches (QTL studies and transcriptome analysis) to identify the new candidate genes *AuxRP* and *Hsp90*. Auxin-regulated proteins are involved in the regulation of the auxin transduction pathway and take part in the response to abiotic stresses. Genes encoding these proteins have also been recognized as stress-responsive (Ghanashyam and Jain 2009; Wu et al. 2012). In our study, the *AuxRP* gene explained up to 16.6 and 15.2 % of the phenotypic variance in chip color AH and after CS, respectively. Moreover, *AuxRP* was expressed only in tubers with light chip color phenotypes.

Heat-shock proteins (Hsp) are a large family of molecular factors involved in many chaperoning functions in plants (Xu et al. 2012). In potatoes, significant differences in protein expression of the chloroplast small heat-shock protein class I, heat-shock protein 70 (Hsp70) and 101-kDa heat-shock protein were detected in tubers between CIS-tolerant and CIS-sensitive cultivars before and after cold storage (Fischer et al. 2013). In microarray experiments, a group of small *Hsp* genes was recognized as cold-linked genes in potato tubers (Bagnaresi et al. 2008). These data are consistent with our finding that a marker derived from the *Chaperone DnaJ* gene encoding the small Hsp40 factor accounted for up to 16.1 and 17.8 % of the chip color after harvest and cold storage, respectively (Table 2). Hsp90 is a highly conserved and essential molecular chaperone that is also involved in abiotic stress responses (Kadota and Shirasu, 2012; Xu et al. 2012). In the present study, we show for the first time increased *Hsp90* mRNA expression and Hsp90 protein expression in the dark chip color progeny bulk samples in comparison with the bulk samples of light chip color phenotypes. *Hsp90* was mapped to potato chromosome VI at 30.0 cM. We did not find a significant association between allelic variation in *Hsp90* and the phenotype (Table 2). However, the gene expression may be regulated by transcription factors or other regulatory elements/proteins located next to or far from the gene that act in *cis* or *trans* configurations (Rockman and Kruglyak 2006). In studies on the response to iron deficiency in soybeans (*Glycine max* L.), only 58 of the 835 (7 %) candidate genes identified in the microarray experiment were mapped within known QTL regions (O’Rourke et al. 2009). Therefore, we suggest that both *AuxRP* and *Hsp90* genes are novel candidate genes capable of influencing the chip color of potato tubers.

## Electronic supplementary material

Supplementary material 1 (DOCX 55 kb)

Supplementary material 2 (DOCX 79 kb)

Supplementary material 3 (DOCX 85 kb)

Supplementary material 4 (DOCX 17 kb)

Supplementary material 5 (DOCX 15 kb)

Supplementary material 6 (DOCX 17 kb)

## References

[CR1] Bagnaresi P, Moschella A, Beretta O, Vitulli F, Ranalli P, Perata P (2008). Heterologous microarray experiments allow the identification of the early events associated with potato tuber cold sweetening. BMC Genom.

[CR2] Chen X, Salamini F, Gebhardt C (2001). A potato molecular-function map for carbohydrate metabolism and transport. Theor Appl Genet.

[CR3] Chomczyński P, Sacchi N (1987). Single-step method of RNA isolation by acid guanidinium thiocyanate-phenol-chloroform extraction. Anal Biochem.

[CR4] Cunningham CE, Stevenson FJ (1963). Inheritance of factors affecting potato chip color and their association with specific gravity. Am Potato J.

[CR5] Douches DS, Freyre R (1994). Identification of genetic factors influencing chip color in diploid potato (*Solanum* spp.). Am Potato J.

[CR6] Draffehn AM, Meller S, Li L, Gebhardt C (2010). Natural diversity of potato (*Solanum tuberosum*) invertases. BMC Plant Biol.

[CR7] Dubos C, Stracke R, Grotewold E, Weisshaar B, Martin C, Lepiniec L (2010). MYB transcription factors in Arabidopsis. Trends Plant Sci.

[CR8] Farre EM, Fernie AR, Willmitzer L (2008). Analysis of subcellular metabolite levels of potato tubers (*Solanum tuberosum*) displaying alterations in cellular or extracellular sucrose metabolism. Metabolomics.

[CR9] Fischer M, Schreiber L, Colby T, Kuckenberg M, Tacke E, Hofferbert H-R, Schmidt J, Gebhardt C (2013). Novel candidate genes influencing natural variation in potato tuber cold sweetening identified by comparative proteomics and association mapping. BMC Plant Biol.

[CR10] Ghanashyam C, Jain M (2009). Role of auxin-responsive genes in biotic stress responses. Plant Signal Behav.

[CR11] Giri J, Vij S, Dansana PK, Tyagi AK (2011). Rice A20/AN1 zinc-finger containing stress associated proteins (SAP1/11) and a receptor-like cytoplasmic kinase (OsRLCK253) interact via A20 zinc-finger and confer abiotic stress tolerance in transgenic Arabidopsis plants. New Phytol.

[CR12] Hamernik AJ, Hanneman RE, Jansky SH (2009). Introgression of wild species germplasm with extreme resistance to cold sweetening into the cultivated potato. Crop Sci.

[CR13] Hubank M, Schatz DG (1994). Identifying differences in mRNA expression by representational difference analysis of cDNA. Nucleic Acids Res.

[CR14] Isherwood FA (1973). Starch-sugar interconversion in *Solanum tuberosum*. Phytochemistry.

[CR15] Jaccoud D, Peng K, Feinstein D, Kilian A (2001). Diversity arrays: a solid state technology for sequence information independent genotyping. Nucleic Acids Res.

[CR16] Jakuczun H, Zimnoch-Guzowska E (2004). Inheritance of glucose content in tubers of diploid potato families. Am J Potato Res.

[CR17] Jakuczun H, Zgórska K, Zimnoch-Guzowska E (1995). An investigation of the level of reducing sugars in diploid potatoes before and after cold storage. Potato Res.

[CR18] Kadota Y, Shirasu K (2012). The HSP90 complex of plants. BBA Mol Cell Res.

[CR19] Kloosterman B, Anithakumari AM, Chibon P-Y, Oortwijn M, van der Linden GC, Visser RGF, Bachem CWB (2012). Organ specificity and transcriptional control of metabolic routes revealed by expression QTL profiling of source–sink tissues in a segregating potato population. BMC Plant Biol.

[CR20] Li L, Strahwald J, Hofferberd H-R, Lübeck J, Tacke E, Junghans H, Wunder J, Gebhardt C (2005). DNA variation at the invertase locus invGE/GF is associated with tuber quality trains in populations of potato breeding clones. Genetics.

[CR21] Li L, Paulo M-J, Strahwald J, Lübeck J, Hofferbert H-R, Tacke E, Junghans H, Wunder J, Draffehn A, van Eeuwijk F, Gebhardt C (2008). Natural DNA variation at candidate loci is associated with potato chip color, tuber starch content, yield and starch yield. Theor Appl Genet.

[CR22] Li L, Paulo MJ, van Eeuwijk F, Gebhardt C (2010). Statistical epistasis between candidate gene alleles for complex tuber traits in an association mapping population of tetraploid potato. Theor Appl Genet.

[CR23] Li L, Tacke E, Hofferbert HR, Lübeck J, Strahwald J, Draffehn AM, Walkemeier B, Gebhardt C (2013). Validation of candidate gene markers for marker-assisted selection of potato cultivars with improved tuber quality. Theor Appl Genet.

[CR24] Lin Y, Liu T, Liu J, Liu X, Ou Y, Zhang H, Li M, Sonnewald U, Song B, Xie C (2015). Subtle Regulation of Potato Acid Invertase Activity by a Protein Complex of Invertase, Invertase Inhibitor, and sucrose nonfermenting1-related protein kinase. Plant Physiol.

[CR25] Malone JG, Mittova VM, Ratcliffe G, Kruger NJ (2006). The response of carbohydrate metabolism in potato tubers to low temperature. Plant Cell Physiol.

[CR26] McCann LC, Bethke PC, Simon PW (2010). Extensive variation in fried chip color and tuber composition in cold-stored tubers of wild potato (Solanum) germplasm. J Agric Food Chem.

[CR27] Menendez CM, Ritter E, Schäfer-Pregl R, Walkemeier B, Kalde A, Salamini F, Gebhardt C (2002). Cold-sweetening in diploid potato: mapping QTL and candidate genes. Genetics.

[CR28] O’Rourke JA, Nelson RT, Grant D, Schmutz J, Grimwood J, Cannon S, Vance CP, Graham MA, Shoemaker RC (2009). Integrating microarray analysis and the soybean genome to understand the soybeans iron deficiency response. BMC Genom.

[CR29] Pereira AS, Tai GCC, Yada RY, Tarn TR, Souza-Machado V, Coffin RH (1994). Effect of selection for chip colour on some economic traits of potatoes. Plant Breed.

[CR30] Pérez-Enciso M, Toro MA, Tenenhaus M, Gianola D (2003). Combining gene expression and molecular marker information for mapping complex trait genes: a simulation study. Genetics.

[CR31] Rockman MV, Kruglyak L (2006). Genetics of global gene expression. Nature.

[CR32] Schreiber L, Nader-Nieto AC, Schönhals EM, Walkemeier B, Gebhardt C (2014). SNPs in genes functional in starch–sugar interconversion associate with natural variation of tuber starch and sugar content of potato (*Solanum tuberosum* L.). G3 Genes Genomes Genet.

[CR33] Sharma SK, Bolser D, de Boer J, Sønderkær M, Amoros W, Carboni MF, D’Ambrosio JM, de la Cruz G, Di Genova A, Douches DS, Eguiluz M, Guo X, Guzman F, Hackett CA, Hamilton JP, Li G, Li Y, Lozano R, Maass A, Marshall D, Martinez D, McLean K, Mejía N, Milne L, Munive S, Nagy I, Ponce O, Ramirez M, Simon R, Thomson SJ, Torres Y, Waugh R, Zhang Z, Huang S, Visser RG, Bachem CW, Sagredo B, Feingold SE, Orjeda G, Veilleux RE, Bonierbale M, Jacobs JM, Milbourne D, Martin DM, Bryan GJ (2013). Construction of reference chromosome-scale pseudomolecules for potato: integrating the potato genome with genetic and physical maps. G3 Genes Genomes Genet.

[CR34] Shin D, Moon S-J, Han S, Kim B-G, Park SR, Lee S-K, Yoon H-J, Lee HE, Kwon H-B, Baek D, Yi BY, Byun M-O (2011). Expression of StMYB1R-1, a novel potato single MYB-like domain transcription factor, increases drought tolerance. Plant Physiol.

[CR35] Sierocka I, Rojek A, Bielewicz D, Karlowski W, Jarmolowski A, Szweykowska-Kulinska Z (2011). Novel genes specifically expressed during the development of the male thalli and antheridia in the dioecious liverwort *Pellia endiviifolia*. Gene.

[CR36] Sierocka I, Kozlowski LP, Bujnicki JM, Jarmolowski A, Szweykowska-Kulinska Z (2014). Female-specific gene expression in dioecious liverwort *Pellia endiviifolia* is developmentally regulated and connected to archegonia production. BMC Plant Biol.

[CR37] Śliwka J, Jakuczun H, Chmielarz M, Hara-Skrzypiec A, Tomczyńska I, Kilian A, Zimnoch-Guzowska E (2012). A new resistance gene against potato late blight originating from *Solanum* *×* *michoacanum* maps to potato chromosome VII. Theor Appl Genet.

[CR38] Śliwka J, Jakuczun H, Chmielarz M, Hara-Skrzypiec A, Tomczyńska I, Kilian A, Zimnoch-Guzowska E (2012). Late blight resistance gene from *Solanum ruiz*-*ceballosii* is located on potato chromosome X and linked to violet flower colour. BMC Genet.

[CR39] Śliwka J, Świątek M, Tomczyńska I, Stefańczyk E, Chmielarz M, Zimnoch-Guzowska E (2013). Influence of genetic background and plant age on expression of the potato late blight resistance gene Rpi-phu1 during incompatible interactions with *Phytophthora infestans*. Plant Pathol.

[CR40] Solomos T, Mattoo AK (2005) Starch-sugar metabolism in potato (*Solanum tuberosum* L.) tubers in response to temperature variations. In: Razdan MK, Mattoo AK (eds) Genetic improvement of Solanaceous crops. Volume I: Potato. Science Publishers Inc., Enfield, pp 209–234

[CR41] Sonnewald U, Kossmann J (2013). Starches-from current models to genetic engineering. Plant Biotech J.

[CR42] Sowokinos JR (2001). Biochemical and molecular control of cold-induced sweetening in potatoes. Am J Potato Res.

[CR43] Stitt M, Hurry V (2002). A plant for all seasons: alterations in photosynthetic carbon metabolism during cold acclimation in Arabidopsis. Curr Opin Plant Biol.

[CR44] Tai GCC, Coleman WK (1999). Genotype × environment interaction of potato chip color. Can J Plant Sci.

[CR45] Urbany C, Colby T, Stich B, Schmidt L, Schmidt J, Gebhardt C (2012). Analysis of natural variation of the potato tuber proteome reveals novel candidate genes for tuber bruising. J Proteome Res.

[CR46] Van Ooijen JW (2006) JoinMap^®^ 4, Software for the calculation of the genetic linkage maps in experimental populations. Kyazma B.V., Wageningen

[CR47] Van Ooijen JW (2009) MapQTL^®^ 6, Software for mapping of quantitative trait loci in experimental populations of diploid species. Kyazma B.V., Wageningen

[CR48] Venkatesh J, Yu J-W, Park SW (2013). Genome-wide analysis and expression profiling of the *Solanum tuberosum aquaporins*. Plant Physiol Biochem.

[CR49] Wenzl P, Kudrna D, Jaccoud D, Huttner E, Kleinhofs A, Kilian A (2004). Diversity arrays technology (DArT) for whole genome profiling of barley. Proc Natl Acad Sci USA.

[CR50] Werij JS, Furrer H, van Eck HJ, Visser RGF, Bachem CWB (2012). A limited set of starch related genes explain several interrelated traits in potato. Euphytica.

[CR51] Wu J, Peng Z, Liu S, He Y, Cheng L, Kong F, Wang J, Lu G (2012). Genome-wide analysis of Aux/IAA gene family in Solanaceae species using tomato as a model. Mol Genet Genom.

[CR52] Xu Z-H, Li Z-Y, Chen Y, Chen M, Li L-C, Ma Y-Z (2012). Heat shock protein 90 in plants: molecular mechanisms and roles in stress responses. Int J Mol Sci.

