# Supplementary material for: Novel candidate genes AuxRP and Hsp90 influence the chip color of potato tubers
Source: Mol Breed. 2015 Nov 18;35:224. doi: 10.1007/s11032-015-0415-1 (PMC4648990; doi:10.1007/s11032-015-0415-1)

**Journal name: Molecular Breeding**

**Novel candidate genes *AuxRP* and *Hsp90* influence the chip color of potato tubers**

Dorota Sołtys-Kalina^1^*, Katarzyna Szajko^1^, Izabela Sierocka^2^, Jadwiga Śliwka^1^, Danuta Strzelczyk-Żyta^1^, Iwona Wasilewicz-Flis^1^, Henryka Jakuczun^1^, Zofia Szweykowska-Kulinska^2^, Waldemar Marczewski^1^*

^1^Plant Breeding and Acclimatization Institute – National Research Institute, Młochów, Platanowa 19, 05-831 Młochów, Poland

^2^ Department of Gene Expression, Institute of Molecular Biology and Biotechnology, Faculty of Biology, Adam Mickiewicz University, Umultowska 89, 61-614 Poznań, Poland

*Corresponding authors: D. Sołtys-Kalina; [d.soltys@ihar.edu.pl](mailto:d.soltys@ihar.edu.pl); +48 22 7299248 ext. 218; fax: +48 22 7299247; W. Marczewski: [w.marczewski@ihar.edu.pl](mailto:w.marczewski@ihar.edu.pl): +48 22 7299248 ext. 215; fax: +48 22 7299247

**Supplementary Fig. 2**  Number of markers and length of genetic map constructed using mapping population 11-40 and the JoinMap ® 4 software (Van Ooijen 2006): distribution of markers and lengths of chromosomes


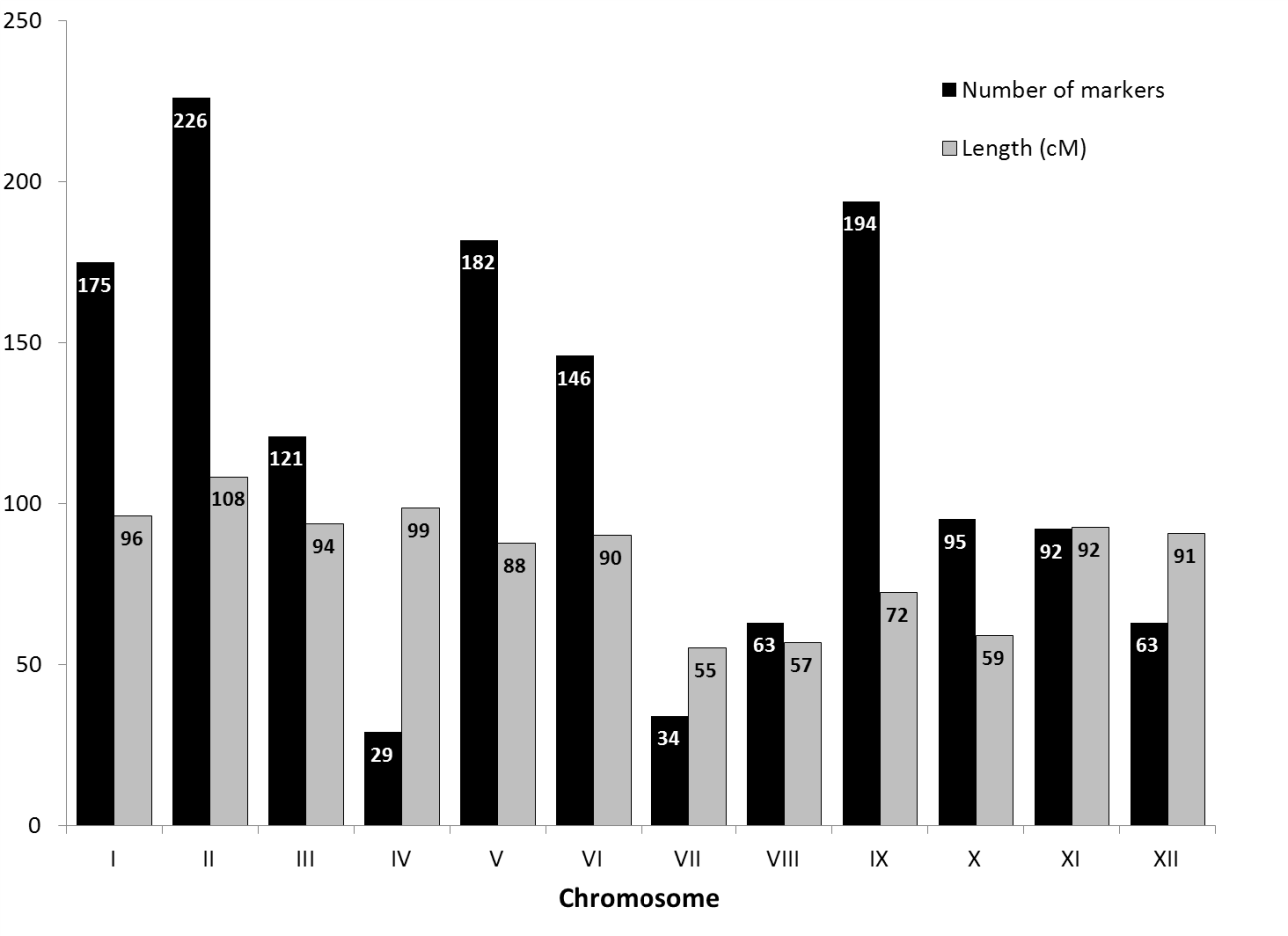

Supplement: Supplementary file 2 — Supplementary material 2 (DOCX 79 kb) [file 11032_2015_415_MOESM2_ESM.docx]
