# Supplementary material for: Novel candidate genes AuxRP and Hsp90 influence the chip color of potato tubers
Source: Mol Breed. 2015 Nov 18;35:224. doi: 10.1007/s11032-015-0415-1 (PMC4648990; doi:10.1007/s11032-015-0415-1)
Supplement: Supplementary file 4 — Supplementary material 4 (DOCX 17 kb) [file 11032_2015_415_MOESM4_ESM.docx]

**Journal name: Molecular Breeding**

**Novel candidate genes *AuxRP* and *Hsp90* influence the chip color of potato tubers**

Dorota Sołtys-Kalina^1^*, Katarzyna Szajko^1^, Izabela Sierocka^2^, Jadwiga Śliwka^1^, Danuta Strzelczyk-Żyta^1^, Iwona Wasilewicz-Flis^1^, Henryka Jakuczun^1^, Zofia Szweykowska-Kulinska^2^, Waldemar Marczewski^1^*

^1^Plant Breeding and Acclimatization Institute – National Research Institute, Młochów, Platanowa 19, 05-831 Młochów, Poland

^2^ Department of Gene Expression, Institute of Molecular Biology and Biotechnology, Faculty of Biology, Adam Mickiewicz University, Umultowska 89, 61-614 Poznań, Poland

*Corresponding authors: D. Sołtys-Kalina; [d.soltys@ihar.edu.pl](mailto:d.soltys@ihar.edu.pl); +48 22 7299248 ext. 218; fax: +48 22 7299247; W. Marczewski: [w.marczewski@ihar.edu.pl](mailto:w.marczewski@ihar.edu.pl): +48 22 7299248 ext. 215; fax: +48 22 7299247

**Supplementary Table 1** CAPS and SCAR markers used in the genetic linkage map

| **Marker** | **Primer sequence (5’→3’)** | **Tm (°C)** | **Amplicon size (bp)** | **Restriction enzyme** | **Marker size (bp)** | **PGSC number** |
| --- | --- | --- | --- | --- | --- | --- |
|  |  |  |  |  |  |  |
| Hsp90 | F: ACATTAATCTCCAACGCCAACA R: ATTTACGATCAACTTCTCATTC | 50 | 670 | *Vsp*I | 550 | PGSC0003DMT400074377 |
| pPt837b | F: TTCCAAAAACCCAGACCAGT R: TACCCCATCCAGACAATAACATA | 55 | 603 | - | 603 | - |
| Myb48g | F: TTGGAGAAATTGGATGAAGA R: GGTTTGAAAAGAAGTGGAAA | 55 | 603 | *Taq*I | 603 | PGSC0003DMT400078151 |
| Myb48d | F: TTGGAGAAATTGGATGAAGA R: GGTTTGAAAAGAAGTGGAAA | 55 | 603 | *Taq*I | 450 | PGSC0003DMT400078151 |
| AuxRP | F: AAGGCGGACGGAAAAGTAATCT R: TTCAAGCAAGTCCATCAAACAAAT | 55 | 660 | *Hinf*I | 490 | PGSC0003DMT400077929 |
| Nod | F: ATGCTCATTTTTGTTTTTGC R: AAGGCCAAATGTCATTACTA | 62 | 542 | *Hpa*II | 490 | PGSC0003DMT400077927 |
| Zfp | F: GGCAGTGGTATATCTGTCTG R: TTGATGATGACAGGTGAGTG | 58 | 594 | *Taq*I | 594 | PGSC0003DMT400083039 |
| Chaperone DnaJ | F: CAATCTCACCAATGCACATC R: TTCTTAGCGATCCACAGAAG | 58 | 785 | *Mnl*I | 360 | PGSC0003DMT400083040 |
| pPt874a | F: ATACACTACACAGCAGCAAGAAAT R: GCCCAACAATCAGCAGTAGAC | 55 | 495 | *Tai*I | 400 | - |
| 965p2 | F: TGCAGGCAGTCATGTTATATGT R: TCCAGCCCAGACAAGTAGAT | 48 | 849 | - | 849 | - |
