# Supplementary material for: Novel candidate genes AuxRP and Hsp90 influence the chip color of potato tubers
Source: Mol Breed. 2015 Nov 18;35:224. doi: 10.1007/s11032-015-0415-1 (PMC4648990; doi:10.1007/s11032-015-0415-1)
Supplement: Supplementary file 5 — Supplementary material 5 (DOCX 15 kb) [file 11032_2015_415_MOESM5_ESM.docx]

**Journal name: Molecular Breeding**

**Novel candidate genes *AuxRP* and *Hsp90* influence the chip color of potato tubers**

Dorota Sołtys-Kalina^1^*, Katarzyna Szajko^1^, Izabela Sierocka^2^, Jadwiga Śliwka^1^, Danuta Strzelczyk-Żyta^1^, Iwona Wasilewicz-Flis^1^, Henryka Jakuczun^1^, Zofia Szweykowska-Kulinska^2^, Waldemar Marczewski^1^*

^1^Plant Breeding and Acclimatization Institute – National Research Institute, Młochów, Platanowa 19, 05-831 Młochów, Poland

^2^ Department of Gene Expression, Institute of Molecular Biology and Biotechnology, Faculty of Biology, Adam Mickiewicz University, Umultowska 89, 61-614 Poznań, Poland

*Corresponding authors: D. Sołtys-Kalina; [d.soltys@ihar.edu.pl](mailto:d.soltys@ihar.edu.pl); +48 22 7299248 ext. 218; fax: +48 22 7299247

W. Marczewski: [w.marczewski@ihar.edu.pl](mailto:w.marczewski@ihar.edu.pl): +48 22 7299248 ext. 215; fax: +48 22 7299247

**Supplementary Table 2** Characteristics of phenotypic distributions of chip color^1^ in population 11-40 after harvest (AH), cold storage (CS) and reconditioning (RC)

| Trait^2^ | N | Population  Mean | Range  of variation |
| --- | --- | --- | --- |
|  |  |  |  |
| AH^a^ | 92 | 5.3 (±0.13) | 3.0-8.7 |
| CS^b^ | 92 | 4.8 (±0.18) | 1.8-9.0 |
| RC^b^ | 87 | 4.1 (±0.18) | 1.8-9.0 |

^1^ average of all years of the experiment

^2^Pearson’s r-values: AH 2011-2012=0.45; AH 2012-2013=0.60; AH 2011-2013=0.30; CS 2012-2013=0.66; RC 2012-2013=0.60

^a^-normal distribution at p<0.05 according to the Shapiro-Wilk test

^b^- deviated from normality at p<0.05 according to the Shapiro-Wilk test
