# Supplementary material for: Novel candidate genes AuxRP and Hsp90 influence the chip color of potato tubers
Source: Mol Breed. 2015 Nov 18;35:224. doi: 10.1007/s11032-015-0415-1 (PMC4648990; doi:10.1007/s11032-015-0415-1)
Supplement: Supplementary file 6 — Supplementary material 6 (DOCX 17 kb) [file 11032_2015_415_MOESM6_ESM.docx]

**Journal name: Molecular Breeding**

**Novel candidate genes *AuxRP* and *Hsp90* influence the chip color of potato tubers**

Dorota Sołtys-Kalina^1^*, Katarzyna Szajko^1^, Izabela Sierocka^2^, Jadwiga Śliwka^1^, Danuta Strzelczyk-Żyta^1^, Iwona Wasilewicz-Flis^1^, Henryka Jakuczun^1^, Zofia Szweykowska-Kulinska^2^, Waldemar Marczewski^1^*

^1^Plant Breeding and Acclimatization Institute – National Research Institute, Młochów, Platanowa 19, 05-831 Młochów, Poland

^2^ Department of Gene Expression, Institute of Molecular Biology and Biotechnology, Faculty of Biology, Adam Mickiewicz University, Umultowska 89, 61-614 Poznań, Poland

*Corresponding authors: D. Sołtys-Kalina; [d.soltys@ihar.edu.pl](mailto:d.soltys@ihar.edu.pl); +48 22 7299248 ext. 218; fax: +48 22 7299247

W. Marczewski: [w.marczewski@ihar.edu.pl](mailto:w.marczewski@ihar.edu.pl): +48 22 7299248 ext. 215; fax: +48 22 7299247

**Supplementary Table 3** Candidate genes obtained from the RDA-cDNA experiment performed in two directions with cDNA from genotypes with a light chip color as a TESTER (L^II^) or as DRIVER (D^II^) after cold storage (CS)

| **Direction of the RDA-cDNA experiment** | **References of cloned amplicons** | **PGSC assignment*** | **Chromosome** |
| --- | --- | --- | --- |
| L^II^ | Conserved gene of unknown function | PGSC0003DMT400066818 | I |
|  | Nuclear RNA binding protein | PGSC0003DMT400066877 | I |
|  | Peptidyl-prolyl cis-trans isomerase | PGSC0003DMT400004125 | I |
|  | Hypothetical gene of unknown function | PGSC0003DMT400093052 | II |
|  | DEAD-box ATP-dependent RNA helicase 52 | PGSC0003DMT400002828 | III |
|  | Mediator complex, subunit Med11 domain containing protein | PGSC0003DMT400060560 | IV |
|  | 60S ribosomal protein L10 | PGSC0003DMT400053166 | VI |
|  | Heat shock protein 90 | PGSC0003DMT400074377 | VI |
|  | 40S ribosomal protein S15 | PGSC0003DMT400042076 | VI |
|  | LeArcA1 protein | PGSC0003DMT400074094 | VI |
|  | ATP synthase epsilon subunit 1 | PGSC0003DMT400068159 | VI |
|  | Vesicle-associated membrane protein | PGSC0003DMT400067225 | VI |
|  | Zinc ion binding protein | PGSC0003DMT400079854 | VII |
|  | Metallocarboxypeptidase inhibitor | PGSC0003DMT400015767 | VII |
|  | Methallothioneine | PGSC0003DMT400023016 | IX |
|  | Hypothetical gene of unknown function | PGSC0003DMT400090811 | X |
|  | 8 sequences not assigned to any gene in the potato genome |  | 6seq-II  1 seq-VIII  1seq-XII |
| D^II^ | Glycine rich RNA binding protein | PGSC0003DMT400001879 | I |
|  | Drm3 | PGSC0003DMT400063589 | I |
|  | DNA-damage-repair/toleration protein DRT111, chloroplastic | PGSC0003DMT400054608 | II |
|  | FRIGIDA | PGSC0003DMT400084922 | VI |
|  | Heat shock protein 90 | PGSC0003DMT400074377 | VI |
|  | Ribosomal protein L28 | PGSC0003DMT400012340 | VI |
|  | Elongation factor 1-alpha | PGSC0003DMT400053547 | VI |
|  | Gibberellin 3-oxidase/ Ribosome biogenesis protein nop10 | PGSC0003DMT400042582/ PGSC0003DMT400065551 | VI |
|  | Metallothioneine | PGSC0003DMT400023016 | IX |
|  | 8 sequences not assigned to any gene in the potato genome |  | 2 seq-I  5 seq-II  1seq-IX |

* assignment of cloned amplicons to genes on the potato genome was verified using Genome Browser v4.03 available at the Potato Genomics Resource (PGSC) platform
